# Accelerating HPV-related cancer elimination – a meeting report

**DOI:** 10.1186/s12919-025-00337-1

**Published:** 2025-08-29

**Authors:** F. Ricardo Burdier, F. Xavier Bosch, Dur-e-Nayab Waheed, Laura Teblick, Mario Poljak, Marc Baay, Silvia de Sanjosé, Marc Bardou, Iacopo Baussano, Irene Man, Eduardo L. Franco, Alex Vorsters

**Affiliations:** 1 Centre for the Evaluation of Vaccination, Vaccine and Infectious Disease Institute, University of Antwerp, Drie Eikenstraat 663, 2650 Edegem, Belgium; 2 Unit of Infections and Cancer (UNIC), Cancer Epidemiology Research Programme, Institut Catala d’ Oncologia-Catalan Institute of Oncology, IDIBELL, Avenida Gran Via 199-203, L’Hospitalet de Llobregat, Barcelona, 08908 Spain; 3 Institute of Microbiology and Immunology, Faculty of Medicine, University of Ljubljana, Ljubljana, Slovenia; 4 P95 Clinical & EpidemiologyServices, Louvain, Belgium; 5 ISGlobal, Barcelona, Spain; 6 CIC1432, CHU Dijon Bourgogne and Université Bourgogne Europe, Dijon, France; 7 International Agency for Research on Cancer (IARC/WHO), Early Detection, Prevention and Infections Branch, Lyon, France; 8 McGill University, Montreal, Canada

**Keywords:** Human papillomavirus infection (HPV), Cervical cancer elimination, Cervical cancer screening, HPV vaccination

## Abstract

The human papillomavirus (HPV) Prevention and Control Board organized a meeting to explore effective strategies for accelerating the elimination of HPV-related cancers, starting from WHO’s cervical cancer elimination campaign targets—vaccination of 90% of girls by age 15, two HPV screenings with a high-performance test for 70% of women between 35–45 years, and 90% treatment and care of women with cervical disease. Nevertheless, the global HPV vaccination coverage remains low (~ 30%), as does screening coverage, with only 24% (48/139) of programmes utilising recommended high-performance tests (such as HPV testing). The meeting explored various strategies, including the extension of vaccination for women at older ages. While vaccination of HPV-positive individuals has demonstrated safety and immunogenicity, further research is required to confirm the potential protective effects and reduced viral transmission among infected populations. Several innovative approaches were discussed, including the HPV Faster strategy, promoting combined HPV vaccination and screening for women up to age 45. This strategy aims to substantially reduce cervical cancer incidence by decreasing future screening needs among HPV-negative women and intensifying follow-up for those already HPV-positive. A variant of this approach, Sweden’s "HPV EVEN Faster," simultaneously vaccinates and screens younger women (ages 23–30), aiming at significantly reducing HPV circulation and effectively reaching underserved populations. Moreover, in resource-limited settings, transitioning to single-dose vaccination emerged as a promising strategy to expand vaccine coverage, as modelled in India, Rwanda, and Brazil. Modelling data reinforced the prioritisation of increasing vaccination coverage and expanding targets in girls up to age 20 as the most efficient strategy to reduce cervical cancers. However, when increasing coverage is challenging, extending vaccination to boys could potentially enhance herd protection. Finally, the discussions underlined that successful “accelerated” HPV elimination strategies must be context-specific, taking into consideration local resources, health system capacities, and socio-economic factors. Political commitment, targeted implementation research, and innovations such as affordable new vaccines and point-of-care tests are key to speed up global progress toward HPV-related cancer elimination.

## Introduction

The human papillomavirus (HPV) Prevention and Control Board is an independent, international, and multidisciplinary group of experts created in 2015 to provide evidence-based guidance and reflection on strategic, technical, and policy issues regarding the implementation and sustainability of HPV prevention and control programmes (https://www.hpvboard.org). The board aims to multiply and disseminate relevant information on HPV prevention and control to a broad array of stakeholders. It contributes to increasing knowledge and awareness to the control of HPV infection, prevention, and screening strategies of HPV-related cancers by organising two meetings every year: a technical meeting covering topics such as vaccine characteristics, vaccine safety, screening technologies and landscape, treatment strategies, the role of healthcare providers in vaccination programmes, and dealing with anti-vaccine messages [1–5]; and a country meeting, covering a strengths, weaknesses, opportunities, and threats (SWOT) analysis of a country or region [6–8].

This report covers the sixteenth HPV Prevention and Control Board meeting (held in June 2024 in Antwerp, Belgium), discussing how to accelerate the elimination of HPV-related cancer. The objectives of the meeting were to:

- Analyse the current status of the World Health Organisation (WHO) Cervical Cancer Elimination Campaign on a global scale and identify opportunities for acceleration of cervical cancer prevention.
- Present and discuss strategies to accelerate the prevention of HPV-related cancers, such as HPV FASTER [9] and HPV EVEN FASTER [10].
- Identify research methodologies for gathering evidence on HPV transmission prevention.
- Understand the feasibility and cost-effectiveness of cancer prevention acceleration strategies.
- Explore the utility of modelling studies in refining and advancing these programmes.
- Analyse the influence of vaccine pricing, dosing, and scheduling on cervical cancer accelerated elimination cost-effectiveness and propose optimisation strategies.
- Discuss methods to measure the goal indicators and cost-effectiveness of accelerated cervical cancer elimination initiatives.
- Deliberate on specific resource-stratified requirements, at the country, level for implementing accelerated cervical cancer elimination.
- Assessment of strategies to accelerate the prevention of HPV-related cancers implementation.
- Understand the next steps and future research/gaps for these strategies.

## Update on the WHO’s cervical cancer elimination goals: *Where are we?*

To achieve the goal of the cervical cancer elimination campaign, i.e., fewer than four new cases of invasive cervical cancer per 100,000 women per year, three targets have been proposed by the WHO [11]:

- 90% HPV vaccination coverage in girls by the age of 15
- 70% screening coverage in women at least twice in their lifetime (around ages 35 and 45), with a high-performance test
- 90% of women diagnosed with cervical disease receiving treatment.

Vaccination has saved 150 million lives over the last 50 years [12]. The HPV vaccine alone can save another 45 million lives, nearly one-third of what expanded programmes on immunisation (EPI) have achieved [12]. However, although 73% of countries (143/194 countries) have introduced HPV vaccination into their national immunisation programme, only 30% of the global population of girls has access to it [13]. Achieving significant access is possible with the implementation of HPV vaccination in low- and middle-income countries (LMICs) with sizeable female populations such as Bangladesh, the Democratic Republic of Congo, Ethiopia, India, Indonesia, Nigeria, Pakistan and Tanzania, which is currently underway [13]. To accelerate progress, these countries need to expand vaccination beyond the typical target population (9–12-year-olds) to include wider age cohorts, reaching millions more girls. In recent years, the demand for HPV vaccines has increased dramatically, outpacing the industry’s capacity to supply them. The shortage meant that several countries, mainly from Africa and Asia have had to wait, and many are still waiting. However, new manufacturers are assisting the campaign with vaccines to address this gap. Furthermore, in 2022, new recommendations introduced a two-dose schedule for all age groups and a one-dose schedule for individuals aged 9 to 20 years [14]. These recommendations have led to a rapid uptake; in June 2024 already 48 countries have adopted the single-dose schedule— representing a third of all countries with HPV vaccination within their immunisation programme. Additionally, several countries have switched to a two-dose schedule for those 15 years and older, further improving efficiency. Implementation research can further boost coverage by examining delivery systems and factors influencing vaccine uptake. A behaviour and social drivers tool is in development to study uptake among parents and adolescents. This tool could play an essential role in effective distribution of resources and increasing overall coverage. To accelerate the process, a prioritisation framework for secondary targets – such as extended age cohorts and gender-neutral vaccination – may be helpful in advising countries with limited resources by providing a rationale for prioritisation.

Alongside efforts to introduce, increase and sustain HPV vaccination coverage (single dose schedule, extended age cohorts, and routine universal HPV vaccination), several initiatives are underway to achieve the second and third elimination targets. Progress has been made towards achieving 70% cervical pre-cancer and cancer screening coverage, particularly in Latin America and the Caribbean [15]; however, the global coverage remains very low. In addition, a great majority of screening programmes do not utilise high-performance HPV molecular tests [16]. To achieve the target, a transition to HPV testing and simple screening algorithms is necessary. In low- and middle-income countries (LMICs), implementing simpler and single-visit strategies, such as “screen-triage-treat” or “screen-and-treat”, are preferable due to challenges in ensuring patient follow-up within existing screening algorithms’ multi-visit care continuum and reducing reliance on pathologists, given the shortage of pathologists and limited access to pathology services in many of these settings. As the focus increasingly shifts towards digital health and artificial intelligence, the potential for leveraging these technologies to expand access to pathology services and automatic colposcopy evaluations in many of these countries stands as an important and necessary development. However, even in larger countries, the reallocation of pathologists within national borders remains challenging. Achieving the cervical cancer elimination goal would be impossible without the use of high-performance HPV tests, given their increased efficacy and standardisation, relative to cytology [17]. However, the role of cytology in triage should be carefully investigated. Integrating HPV testing with self-collected samples and ensuring access to ablative treatment for those who require it are both relevant interventions to enhance the impact of cervical cancer screening. Globally, 139 countries have started cervical screening programmes, 48 use HPV-based screening, and 17 allow self-collection, which implies a wide diversity of screening algorithms [16]. This variation highlights the need for a robust framework to evaluate these algorithms objectively.

The WHO is launching living guidelines for cervical cancer screening (https://www.who.int/news/item/08-11-2022-who-announces-the-development-of-recommendations-for-screening-and-treatment-to-prevent-cervical-cancer-in-women-living-with-and-without-hiv), which are continually updated as new evidence is emerging in five main priorities: HPV extended genotyping, self-collection, approaches for women living with HIV, artificial intelligence (AI) for analysis of cervix images, and HPV point-of-care (POC) testing. To support implementation, Target Product Profiles (TPPs) for HPV tests—defining minimal and preferred parameters as shown in Table 1—were developed through Delphi consensus and public consultation [18]. These include separate TPPs for laboratory and POC tests. While TPPs clarify targeted HPV types, challenges remain. Many tests still target low-risk HPV types, contradicting guidelines which recommend screening for only 12 high-risk (carcinogenic) types. Despite discouragement, clinicians often use such tests. With more than 260 distinct commercial HPV assays (and 511 test variants) available globally (only 20 clinically validated and one near-POC) [19, 20], the WHO’s lack of regulatory authority hinders enforcement. This underscores the need for alignment with TPPs to improve clinical accuracy and reduce market fragmentation.

**Table 1:** WHO target product profiles (TPPs) for HPV screening tests

	**Minimal Characteristic**	**Preferred Characteristic**
**Specimen Type**	– Self- and provider-collection for LAB tests – Only self-collection for POC tests	– Self- and provider-collection for LAB & POC tests
**Genotyping Spectrum**	– 8 most oncogenic HPV types^a^ included for LAB & POC tests	– 12 most oncogenic HPV types^b^ included for LAB & POC tests
**Result Output**	– 2–3 genotyping groups for LAB tests – Pos/Neg output for POC tests	– 4 genotyping groups for LAB tests – 2–3 genotyping groups for POC tests
**Cost per reportable result (all inclusive)**	– ≤ US$8 for LAB & POC tests	– ≤ US$5 for LAB tests – ≤ US$3 for POC tests

*LAB *Laboratory test, *POC *Point-of-care test

^a^8 most oncogenic HPV types (16, 18, 45, 33, 58, 31, 52, 35)

^b^12 most oncogenic HPV types (16,18, 45, 33, 58, 31, 52, 35, 59, 39, 51, 56). Table adapted from [18]

A survey examining the inclusion of cancer care in health benefits packages worldwide showed that only 25% of low-income countries and 54% of lower-middle-income countries include treatments like radiotherapy [22]. Packages of essential services, including screening and treatment of invasive cancer, have been developed and endorsed by strategic partnerships, such as with the Islamic Development Bank or the International Agency for Atomic Energy.

Although progress is being made, it needs to be accelerated through innovative approaches, stronger partnerships, and coordination. Local stakeholder leadership is driving improvement, increasing capabilities, and addressing inequalities. However, progress can only be achieved if we measure impact and outcomes.

## Insights into HPV natural infection and transmission dynamics

### Research insights on latency, immune control and subclinical infection

HPVs are a diverse group of small DNA viruses (± 8,000 bp) targeting epithelial tissues capable of causing a short-term or long-term infections [22]. A distinctive feature of viruses causing long-term infections is their ability to establish a reservoir that persists even when no viral particles are actively produced. For HPVs, the reservoir is the basal layer of epithelial cells, the basal cells [22].

While HPV lesions can regress or be treated, the viral genome is not eradicated, allowing the possibility of later reactivation. This challenges the belief that new lesions arise solely from new infections; emerging data suggest that they may also result from the reactivation of latent virus or changes in viral activity [22, 22]. These findings support the hypothesis that infection clearance reflects immune control rather than complete viral elimination. Results from animal models suggest that following inoculation of an HPV-naïve animal, lesions rapidly develop. Upon immunosuppression, HPV DNA was detected at levels consistent with a productive infection, although microlesions were rarely seen given the limited follow-up time [22]. Studies in humans measuring episodic detection of HPV have shown that redetection of HPV DNA is common when samples are taken every 3–4 days for 16 weeks [22] or every 4–6 months for a median of 6.5 years [22]. This could further support the finding that recurrent HPV infections occur more frequently in women who naturally developed HPV16 antibodies [22], raising the possibility that HPV16 antibodies are most likely serological markers for latent or past infections.

**Fig. 1:**
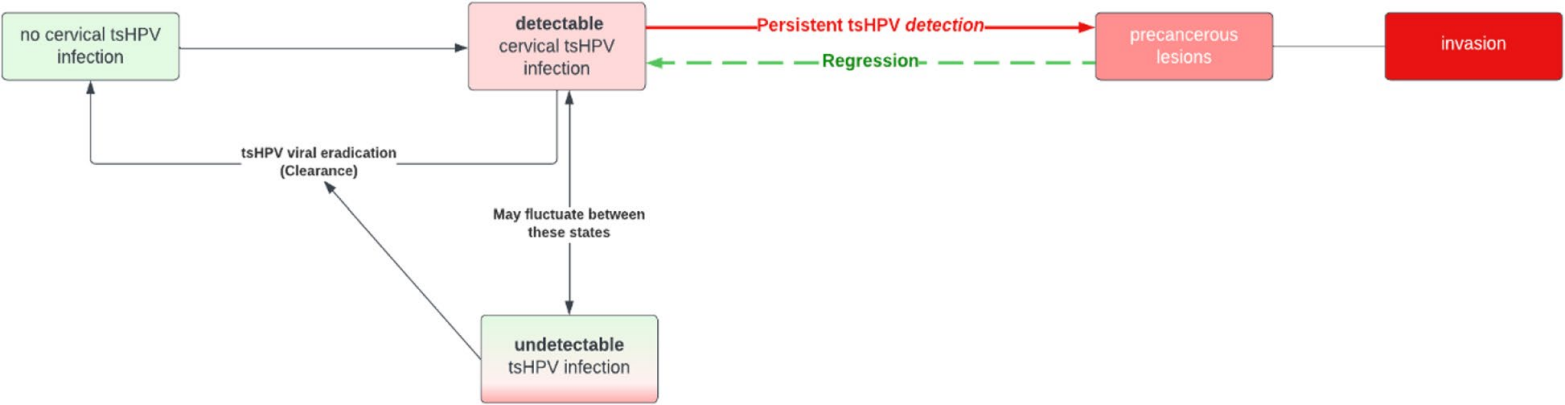
Schematic model of the natural history of human papillomavirus infection and cervical cancer. Adapter from Gravitt et al. [22]. tsHPV – Type-specific Human Papillomavirus

The HITCH study, a cohort study of newly formed heterosexual partners to understand transmission, estimated that 43% of incident type-specific HPV infections (544 events among 849 participants) were non-attributable to recent sexual transmission [22], suggesting reoccurrence through virus reactivation. Furthermore, first HPV detection or recurrent detection does not lead to a difference in low- or high-grade disease prevalence [22]. Based on these findings, the HPV natural history may need to be adapted such as proposed in Fig. 1.

The primary mode of action of the HPV vaccines is the induction of neutralising antibodies that prevent entry of viral particles into the basal epithelial cells and thereby infection. This is also the case with natural infection; however, the immune response is suboptimal in preventing infection compared to that after vaccination. However, current vaccines are prophylactic, which is why vaccination is recommended at younger ages when prior exposure is less likely. Nevertheless, these findings suggests that HPV reactivation later in life is a plausible event and could have important implications for vaccination strategies. In particular, they suggest a rationale for considering HPV vaccination in adult women, as immunization may help protect against the reactivation of previously acquired HPV genotypes. Although current HPV vaccines are prophylactic and thus primarily recommended before exposure, vaccination of HPV-positive individuals has been shown to be as safe and immunogenic as in HPV-negative individuals [22]. Moreover, it may help reduce the infectivity of viral particles, thereby lowering the risk of transmission or autoinoculation [3]. Further research on HPV persistence, reactivation and its interaction with vaccine-induced antibodies is needed to support these strategies.

Most studies investigating HPV natural infection largely rely on systemic serologic biomarkers, while studies using local biomarkers (scrapes or brushes) could be minimally invasive sampling methods that disrupt mucosal homeostasis and bias results. By contrast, first void urine (FVU) offers a non-invasive alternative, capturing secretions from the uterine, cervical, and vaginal epithelia that have accumulated between the labia minora. Biomarkers such as HPV DNA and antibodies remain stable for a considerable amount of time, making FVU a promising tool to study HPV infections, vaccination follow-up, cervical cancer screening, and epidemiological research [22].

In a recent study, paired FVU and serum samples were collected from 58 women before vaccination, one month after receiving three doses of the 9vHPV vaccine, and approximately 3.5 years post-vaccination. Detectable antibodies were present in 95% to 100% of FVU samples at one month and persisted in 84% to 100% after 3.5 years, varying by HPV vaccine type [22]. Despite lower antibody concentrations in FVU compared to serum, significant positive Spearman correlations were observed between the two sample types, with correlation coefficients ranging from 0.36 to 0.82 depending on HPV type and sampling time [22]. Interestingly, naturally induced antibody responses prior to vaccination were observed in up to 16% of the women, indicating that local infection-induced immune responses can be detected using this sample type. Additionally, another study revealed that anti-HPV16 antibodies in FVU samples maintain their neutralising capacity and can be detected up to ten years after vaccination [22]. However, optimized detection and purification protocols remain essential. Additionally, purified HPV virions from FVU are currently being explored in vitro to assess whether vaccination reduces viral transmission and autoinoculation [22, 22].

### What do we know from HPV vaccination beyond its prophylactic effect?

When examining the protective effects of the HPV vaccine beyond prophylaxis, the case of recurrent respiratory papillomatosis (RRP) provides notable insights. RRP is a rare, benign, yet chronic condition caused by HPV types 6 and 11. It is characterised by recurrent wart-like growths predominantly affecting the larynx. The disease exhibits a trimodal age distribution: juvenile-onset around age five, followed by adult-onset occurring between 30–40 years and again around 60 years. Juvenile onset RRP (JoRRP) is more aggressive and prevalent than the adult types, imposing a significant clinical burden. While vertical transmission from mother to child has been suggested, the exact mode of viral transmission remains unclear, particularly since caesarean delivery has not been observed to impact infant infection rates [22]. A significant decrease in incidence was observed in countries with widespread access to HPV vaccines that cover HPV6/11 [22, 22].

Managing RRP remains challenging due to the lack of a definitive cure and the frequent recurrence of symptoms in the aggressive type. Surgical debridement iscurrently the only option to manage recurrences, aiming to maintain airway patency and preserve voice while minimising scar tissue. However, repeated surgical intervention places a significant financial burden on the healthcare system and caregivers. Approximately 50% of affected children have more than ten procedures, with 7% having more than 100 operations during their lifetime and one-third requiring surgical care into adulthood.

Emerging evidence from the off-label use of HPV vaccines targeting HPV6/11 may offer options beyond surgical debulking. Although large-scale randomised clinical trials are impractical due to the rarity and clinical variability of RRP, data from case series where RRP patients were vaccinated have shown increased inter-surgical intervals, leading to fewer lifetime surgery procedures [22]. Vaccination appears safe for children who would typically receive it from the age of nine, and immunising mothers positive for HPV6/11 during pregnancy, or earlier vaccination in children, may help mitigate disease severity, prevent viral re-infection, and reduce long-term morbidity [22]. Despite the low level of evidence due to small sample sizes and potential biases, the findings align with the natural history of this disease that goes into remission in these children.

Although persistent high-risk HPV infections clearly pose the greatest risk for precancer and cancer development—while negative test results correspond to minimal risk—several critical questions remain unresolved. Current studies often rely on the interpretation of HPV results as a proxy for true infection status. Although HPV testing is highly effective for predicting cervical cancer risks, the interpretation of HPV tests to accurately understand the dynamics of natural infection remains suboptimal. Additional limitations include left truncation (unknown prior HPV history), interval sampling (undetermined infection status between visits), right truncation (post-study status), and insufficient measurement of local mucosal immunity. These factors make it difficult to distinguish between genuine viral clearance and immune-controlled undetectable infection. Furthermore, it is not yet clear whether newly detected HPV represents new acquisition, reinfection, reactivation of latent infection, recent deposition, or autoinoculation, nor how these different pathways affect long-term cancer risks. Hence, it is important to recognise that while L1 protein is present during productive infection, its role may be less prominent under immune-controlled infections, suggesting that alternative proteins may be necessary for targeting different clinical outcomes. Addressing these gaps is critical for informing future research and for refining both vaccination and screening strategies aimed at optimising, preventing and managing HPV-related precancer and cancer.

Investigating why certain individuals do not clear HPV infection is of critical importance. Although vaccine-induced protection against HPV is predominantly driven by humoral responses [22], cellular immunity—including interactions between T and B lymphocytes and the production of various cytokines—also appears to play an influential role [22]. Accordingly, a comprehensive understanding of the tissue microenvironment becomes essential. Research on therapeutic HPV vaccination demonstrated that individuals with HPV16-driven CIN2 + exhibit robust T-cell responses in peripheral blood, but these responses are not observed in the epithelial compartments [22]. This discrepancy underscores how a dysregulated tissue microenvironment may impede effective immune clearance. Moreover, evidence presented suggests that the virus may have developed mechanisms to establish latency, with the potential to reactivate once the host immune response diminishes.

Evidence from the Ludwig-McGill study suggests that naturally occurring neutralising antibodies offer limited protective benefits [22]. At the same time, many participants in these trials likely already had latent infections, implying that vaccination may be contributing to the suppression of viral reactivation. To test this hypothesis, future investigations could examine vaccinated populations with known serological profiles, particularly among individuals who tested positive for HPV16 at enrolment, to determine whether the vaccine mitigates recurrence. Additional evidence in longitudinal cohorts may further illuminate these processes. If a link exists between immune control and advancing age, one might observe increased HPV prevalence among women aged 50–85 who are no longer sexually active. Analysing subsets of these individuals according to HLA patterns —especially those associated with weaker immune responses—could yield further insights. Notably, an upsurge in HPV positivity with advancing gestational age has been reported [22, 22], suggesting that pregnancy-related hormonal changes or altered immune responses may contribute to increased HPV acquisition or reactivation [22].

Finally, the potential application of HPV vaccination as an adjunct therapy for women with preneoplastic lesions warrants careful consideration. Theoretically, the HPV vaccine is safe, it seems reasonable to consider vaccinating high-risk individuals to reduce the risk of reinfection, however, further data supporting this hypothesis is needed. Surgical interventions like cone biopsies and LEEP can create mucosal entry points for viral re-infection, yet neutralising antibodies from vaccination might prevent HPV from establishing itself in these sites. Further research, particularly in immunocompromised populations, is essential to refine our understanding of the interplay between HPV biology, host immunity, and vaccine-induced protection.

## Accelerating elimination of cervical cancer: transforming current insights into equitable strategies for improving coverage globally

### Understanding the concept of HPV FASTER, EVEN FASTER and the way forward

While the priority remains vaccination of girls aged 9–14 years, many countries now extend this to the vaccination of young women up to the age of 26 [22]. Building upon this framework, the HPV FASTER strategy proposes extending vaccination to women up to ages 40–45, contingent on logistical and economic factors, as well as each country’s capacity to effectively reach its target population. Although its assumption aligns with evidence-supported dogmas of HPV immunology and epidemiology, HPV FASTER remains a theoretical proposal requiring empirical validation. By integrating vaccination with both routine and opportunistic screening visits, this strategy proposes that women can receive the vaccine regardless of their HPV test results. If HPV-negative women are vaccinated, this group of women should need less intensive screening afterwards, which reduces costs through a reduction in the number of visits. Eventually, the risk of those women to develop cervical cancer in the future should be very low. The HPV-positive women who are vaccinated after infection, expected to be between 20 and 30% of the population, will require active follow-up to avoid progression. However, vaccination may reduce transmission. Furthermore, including boys in the equation would increase the programme’s resilience and boost herd protection. Additionally, vaccinating the partners of HPV-positive women could further control viral circulation. While this strategy warrants further discussion, a robust public health approach is essential to assess its effectiveness in reducing viral transmission. Herd protection would represent a key added value of interrupting transmission, which could be attributed to at least two components: first, the protection afforded by vaccinating HPV-naive women—who, by preventing infection, reduce the background risk of transmission per sexual encounter; and second, the potential reduction in transmissibility among vaccinated, previously infected women [9].

The EVEN FASTER concept expands on the FASTER strategy to reduce HPV reproductive rates. In infectious disease epidemiology, the basic reproductive number (R_0_) quantifies the transmission potential of a pathogen. For HPV—a primarily sexually transmitted infection—R_0_ is strongly influenced by sexual behaviour patterns, acquisition rates, and viral persistence dynamics. Current models estimate an R_0_ between 2 and 3, though validation across diverse populations and age groups remains critical. Achieving herd protection against HPV requires vaccination coverage of approximately 50%, facilitated by neutralising antibodies that block viral binding to the basal membrane and cellular entry, thereby preventing infection. These antibodies may also reduce autoinoculation or transmission by neutralising shed virions in HPV-positive individuals [22]. HPV EVEN FASTER strategy relies on two critical pillars: HPV-based screening and HPV vaccination. A single HPV test can determine the long-term risk of cervical cancer. In several countries where HPV-based screening trials have been performed, e.g., the Netherlands [22], Sweden [22], and US [22], individuals who tested HPV-negative had a near-zero risk of developing cervical cancer [22]. Since 2008, Global HPV LabNet proficiency panels have tracked improvements in laboratory capacity for HPV testing. In 2008, only 35 of 81 laboratories (43.2%) accurately identified HPV in all samples. By 2023, 123 of 141 laboratories (86%) achieved full accuracy, demonstrating that testing quality has significantly improved worldwide.

In Sweden, before the introduction of HPV vaccination, the incidence of HPV16 among women peaked at the age of 17 years. To assess the potential impact of HPV vaccination at the population level, the basic reproduction number (R_0_) was calculated for different age groups. R_0_ represents the average number of new infections caused by a single infected individual. An R_0_ below 1 indicates that the infection will gradually disappear, as each infected individual infects fewer than one other person on average. The study found that R_0_ was 1.3 for women aged ≥ 25, 1.1 for those aged ≥ 30, and 0.4 for those aged ≥ 35. This suggests that HPV infections will rapidly decline among women older than 35 if new infections from younger populations are prevented. Furthermore, the R_0_ value of 1.1 observed in the group aged ≥ 30 indicates that there is still an opportunity for targeted vaccination to reduce R_0_ below 1, potentially leading to the elimination of HPV-vaccine-type circulation within the Swedish population [10].

These population-level observations align with vaccine efficacy studies, reinforcing the importance of reaching key age groups while they are still HPV-naive. In a pre-licensure study of the bivalent HPV vaccine (2vHPV), researchers observed high efficacy—about 90% against HPV16/18 infection and up to 87% prevention of CIN3 + lesions—but only when women were confirmed HPV-negative at baseline [22]. In contrast, the protective effect was not statistically significant in the intention-to-vaccinate group, where HPV status at vaccination was not assessed. These findings highlight the importance of identifying HPV status at the time of vaccination in older age groups. Modeling studies show that eliminating HPV16 requires 90% coverage if only girls are vaccinated, whereas HPV18 can be eliminated with 80% coverage. With gender-neutral vaccination, HPV16 elimination can be achieved at 70% coverage. While catch-up vaccination reduces infections, it cannot fully eliminate them [22].

The EVEN FASTER campaign invites women between 23 and 30 years to concomitant HPV vaccination and HPV screening. Even a 30% coverage of this intervention is predicted to result in a considerably faster decline in the incidence of infection. This strategy has two goals: to stop the circulation of HPV as soon as possible and to offer HPV screening to those who may have been infected before circulation was stopped and who may have developed precursors. Screening is an integral part of the elimination strategy. All women with a risk of cervical cancer of about 0.1% over five years in Sweden are invited to one of 633 enrolling sites all over the country. This campaign is also an effective way to reach women with insufficient screening across Sweden. With this strategy, it should be possible to achieve a reduction of cervical cancer incidence below 4 per 100,000 in the next three years.

When aiming for cervical cancer elimination, catch-up vaccination in the age groups that are still transmitting the infection with a reproductive rate of more than one, combined with a large-scale effort to reach the non-attenders with HPV screening, is the fastest way forward.

During the meeting, several countries such as Mexico, Israel, Bhutan and Costa Rica presented their HPV vaccination structure and plans to expand access and coverage. In this section, we focus on countries experiences from Mexico and Costa Rica given the relevance for accelerating elimination of cervical cancer in relevance with EVEN Faster concept.

#### Mexico

Mexico was among the early adopters of HPV testing as a primary screening method, initially implementing it nationwide in place of cytology. However, the country later reverted to cytology due to practical challenges, including issues related to implementation logistics, cost, infrastructure limitations, and integration with existing health programmes [22]. Efforts are currently underway to reintroduce HPV testing and potentially streamline HPV vaccination, offering an opportunity to evaluate the applicability of the HPV FASTER concept in a real-world setting. A study in Tlalpan, a neighbourhood in Mexico City, was proposed to evaluate the HPV FASTER concept. As part of the regular programme, using HPV as a primary sample for screening procedures, urine, vaginal and cervical samples were collected from 3000 women aged between 25 and 45. The women were randomised and vaccinated; one-third with the 2vHPV vaccine, one-third with the 4vHPV vaccine, and one-third were non-vaccinated controls. These women will be screened again to look at the incidence of CIN2 +. Vaccination will be offered to the control groups at the end of the study [22]. One early observation is that self-sampling has become much more acceptable; women are less afraid they are not sampling correctly. Completion of the study is expected by mid-2025.

#### Costa Rica

Costa Rica, a small country of about 5 million people, implemented HPV vaccination in 2019 for a single birth cohort of girls through a school-based programme using a two-dose regimen. High vaccine uptake was achieved until the pandemic caused a decline, but uptake is now recovering. Cervical cancer screening has been an opportunistic programme since 2006, using conventional cytology. In 2023,a population-based HPV-based screening programme was introduced. Although cervical cancer incidence has declined over time, it remains high at approximately 20 per 100,000. Current efforts prioritise vaccinating young girls to reduce future cases. However, without timely additional interventions, the opportunity to further accelerate elimination may be lost as this cohort ages. Therefore, prompt action is essential to maximise the impact of supplementary measures. One key question is what additional value-added opportunities can be introduced quickly, beyond the current efforts, to further reduce incidence and accelerate elimination?

Several single-dose HPV vaccine studies are ongoing in Costa Rica, including the PRISMA study [4], which provides an opportunity for HPV FASTER. This study evaluates the efficacy of a single-dose HPV vaccine in adult women. Initially, 5500 women were enrolled, with cervical samples collected to rule out prevalent HPV16/18 infections. Three months later, at the vaccination visit, these women were randomised to one of three arms: a single dose of the nonavalent HPV vaccine, a single dose of the bivalent vaccine, or a control arm receiving 1 dose pf diphtheria toxoid/tetanus toxoid/acellular pertussis vaccine adsorbed vaccine. Cervical samples were again collected to rule out prevalent HPV infection.

These women, who will be 21 to 33 at the end of the three-year follow-up, are monitored every six months for virologic and immunologic endpoints. A safety exit procedure ensures that participants leave the study with an average risk of cervical cancer. Adding one additional post-screening cycle could be highly informative, particularly given the limited sample size, which restricts the ability to look at histological endpoints. Additional time would allow new acquisitions, time to clear, and time to grow into lesions. Data from Costa Rica could be more broadly applicable to other countries, perhaps in Latin America or other middle-income countries, than the data from Sweden. In parallel, Costa Rica’s screening programme has recently changed to HPV-based testing, incorporating genotyping and cytology for triage negatives and colposcopy for positives. By testing 250,000 women annually, the entire country—about 1.2 million women— should be covered in five years. The necessary infrastructure and equipment are already in place, so by 2030, all elimination goals should be met.

### Toward faster cervical cancer elimination in low-resource settings: modelling single-dose, gender-neutral, and extended-age vaccination approaches

The opportunity to switch to single-dose vaccination allows the second dose to be reallocated to vaccination of others. The question then becomes how best to allocate the resources saved. Modelling was performed in three LMICs with varying cervical cancer burdens and timelines for HPV vaccination introduction: India, Rwanda, and Brazil. Among these, Rwanda has the highest cervical cancer burden, followed by India and Brazil.

The reference is a continuation of the two-dose strategy. Then, a switch to single-dose vaccination in 2025 combined with different allocation strategies. These strategies included (1) a one-off catch-up campaign for older female cohorts up to 30 years in 2025, (2) improving routine vaccination coverage, and (3) transitioning to routine gender-neutral vaccination. For each country, the number of doses and resources saved were calculated, and the different reallocation strategies were ranked by dose efficiency. The total impact of each strategy was assessed, including the potential to reach cervical cancer elimination [22].

In India, where vaccination has not yet been introduced, a 50% coverage assumption would save around 55 million doses. The most dose-efficient reallocation strategy is catch-up vaccination for females up to 20 years old. The next most dose-efficient strategy would be to increase the routine coverage in girls from 50 to 90%. Catch-up vaccination for females aged 21–30 and gender-neutral vaccination have the lowest dose-efficient. A sensitivity analysis with lower efficacy and shorter duration of protection of single-dose vaccination still increased the total impact of reallocation of doses [22]. In India, the elimination targets can be achieved by reaching 90% coverage among female cohorts; however, if this target is not met, vaccinating male cohorts could compensate, though at the expense of requiring additional doses to reduce cervical cancer incidence below 4 per 100,000 ASR.

Rwanda introduced HPV vaccination for girls in 2011, so most cohorts up to 25 years old have already been vaccinated. Catch-up for women aged 26–30 has the highest dose efficiency. Gender-neutral vaccination will require slightly more doses than those saved by moving to a single dose, but it will increase the impact, even in the worst-case scenarios of single-dose waning protection. However, because of the cervical cancer burden, even with adding vaccination of boys and reaching 100% coverage, the elimination threshold would not be reached [22]. Switching to the nine-valent vaccine and establishing a screening programme may help reach the elimination target and be more effective than gender-neutral vaccination.

In Brazil, some northern states have a higher cervical cancer burden and lower vaccination coverage than the rest of the country. Improving the routine coverage in those states to match the national coverage rate is the most efficient strategy and improves equity. This strategy only needs around 3% of the doses saved by moving to single-dose vaccination. The remaining doses can be used for catch-up vaccination of women aged 26–30, further increasing impact, even in scenarios with waning immunity [22]. The elimination targets can be reached, even in the high-burden states, by increasing coverage for girls, supported by the vaccination of boys.

The move to single-dose vaccination with strategic resource allocation is a highly efficient way to increase the overall impact of vaccination, even in the worst-case scenario of waning immunity [22]. This approach could accelerate the progress towards elimination and make access to vaccination more equitable. Some fine-tuning will remain necessary to reflect the situations in other countries.

### Optimal HPV vaccination strategies in LMICs to reach cervical cancer elimination

Prioritising HPV vaccination strategies requires different approaches depending on the context. When aiming for elimination in an unrestricted context, the goal is to maximise health benefits, leading to an absolute reduction in cancer incidence over time. However, when vaccine supply is constrained, the focus shifts to maximising health benefits at minimal cost. In this case, efficiency is measured by the number needed to vaccinate to prevent one case of cervical cancer (NNV). When there are budget constraints, the objective is to maximise health benefits while minimising the required doses; in this case, cost-effectiveness is measured as the cost per disability-adjusted life years (DALY). Mathematical modelling was used to assess various factors and identify the most efficient HPV vaccination strategies in LMICs [22]. These included varying the target population (girls-only, girls and boys, or different adults age ranges through multiple-age cohort (MAC) vaccination), the number of doses (2-dose or 1-dose vaccination, the latter with lifelong duration of protection, or mean protection duration of 30 years), and vaccination coverage [80% as the mean coverage in LMICs, 40%, 70%, or 90% (the cervical cancer elimination target)]. Using these data, an efficiency frontier was calculated, ranking all strategies from the lowest to the highest incremental NNV, starting with girls-only routine vaccination at 9 years old as the initial strategy. At 80% coverage, extending single-dose vaccination to girls aged 10–14 and 15–20 was almost equally effective (NNV 48 vs 64), while two-dose vaccination of women 21–25 years of age and vaccination of boys was less effective (NNV 369 vs 511, respectively). While MAC vaccination of older girls accelerates cervical cancer reduction with limited numbers of extra doses, routine vaccination of boys maximises cervical cancer reduction but doubles the number of doses used [22].

When routine vaccination coverage is lower (40% or 70%), vaccinating girls up to age 20 remains the most efficient approach. The next best strategy is to increase single-dose coverage among these girls by even just 10%, regardless of whether starting at 40%, 70%, or 80% coverage. For instance, doubling vaccination coverage among girls from 40 to 80% leads to three times more cervical cancers averted compared to vaccinating boys at 40% coverage. If it is unfeasible to increase coverage, vaccinating boys can be an efficient use of HPV vaccines as it can increase the protection of girls through herd immunity. However, as sociodemographic and regional factors may influence vaccine uptake, vaccinating boys may disproportionately protect the sexual partners of vaccinated females, thus limiting the incremental benefit. Using 30 years of protection instead of lifelong protection does not change the prioritisation; vaccinated girls and women are protected during their peak ages of sexual activity. When considering cervical cancer burden, in settings with moderate to very high burdens, vaccinating women up to age 25 should be prioritised before vaccinating boys. In contrast, in low-burden settings, catching up of older women (26–30) should take precedence over boys. Equity considerations are also crucial in prioritisation. About 85% of cervical cancers worldwide occur in LMICs, yet only 26% of women in LMICs are ever screened versus 83% in HICs. Similarly, inequalities have the potential to increase due to inequitable vaccine distribution: in LMICs, 16% of girls are vaccinated versus 59% in HICs.

### Impact of catch-up HPV vaccination on cervical cancer incidence in Kenya in the context of moderate HIV prevalence

HIV-positive women have a higher risk of acquiring HPV infection. The risk is inversely associated with the CD4 cell count, while antiretroviral therapy lowered HPV acquisition, increased clearance, and reduced precancer progression, likely via immune reconstitution [22]. Population-level effectiveness of catch-up HPV vaccination at different levels of HIV prevalence is currently unknown.

For modelling purposes, moderate and high HIV prevalence settings in Kenya were explored. Several interventions were modelled. The baseline strategy was no vaccination, allowing the impact of the different vaccine strategies to be assessed. The first scenario reflected the current Kenyan vaccination strategy: one-dose vaccination at 90% coverage of girls aged 10. The second scenario looked at the MAC of girls aged 10–14 at 90%. The third scenario examined the previous plus one year of catch-up vaccination of 15–24-year-old women at 50% coverage. The fourth scenario was similar to the third, but with 80% coverage of the 15–24-year-old women. The final scenario was the HPV FASTER scenario, with a one-year catch-up of women aged 15–44 at 80% coverage.

All scenarios showed accelerated cervical cancer elimination, regardless of HIV prevalence, with incidence rates ranging from 9 (current vaccination strategy) to 4 (HPV FASTER) per 100,000 women [22]. HPV vaccination had little impact on HIV acquisition. To achieve cervical cancer elimination, even in a scenario with HPV vaccination, screening and treatment will remain necessary. Finally, looking at cost-effectiveness, any single-dose scenario dominates the two-dose scenario. Among the single-dose scenarios, the scenario with 90% routine vaccination coverage plus catch-up vaccination of girls/women between ages 10–20 at 50% coverage is the most cost-effective, with a price of $2742 per averted case of cervical cancer. The money saved could go into other programmes, e.g., rotavirus vaccination.

### Current and upcoming projects for eliminating cervical cancer in at-risk populations

This section outlines projects aimed at strengthening national cervical cancer screening programmes worldwide. While such programmes generally demonstrate effectiveness—albeit to varying degrees—they provide a foundational system to prevent, control, and treat cervical cancer. Nonetheless, reaching the most vulnerable populations, particularly migrants, remains a critical challenge. Within the European Union, the issue of migrant health is politically sensitive, often complicating policy decisions and resource allocation. Despite these hurdles, several initiatives in Europe are actively working to include underserved communities, underscoring the importance of equitable access to screening and care for all.

One such initiative is the Risk-based Screening for Cervical Cancer project (RISCC), an EU-funded, multidisciplinary consortium of HPV researchers. RISCC aims to develop and evaluate the first risk-based screening programme for cervical cancer, provide open-source implementation tools, and contribute to the elimination of cervical cancer in Europe. The project combines RHEA transmission model and the PROgression ModEl of The HPV infEction (ProMetHeoS) progression model to simulate various screening scenarios, including those of LMICs. The model stratifies risk by HPV genotype, age group, and vaccination status and can be adapted for subpopulations, e.g., migrants.

Another initiative is the EVEN FASTER project, as explained in Sect. 4.2, which simulates the Swedish vaccinated birth cohorts (1994–1998) of women targeted by the nationwide trial. By employing the EVEN FASTER intervention—concomitant HPV vaccination and HPV screening, with an adherence of 30% or 90%— it can be shown that the programme truly works faster [22]. The model, now calibrated, is being adapted to subpopulations, including migrants, using data from the Netherlands, Spain and Sweden.

In France, the number of cervical cancer cases decreased from 3900 in 1990 to 2900 cases in 2018, with the incidence falling from 10 to 6 per 100,000. However, the decline has plateaued, indicating that further efforts are needed, particularly for the high-risk populations. In high-income countries, vulnerable populations may include socioeconomically disadvantaged women, women living with HIV, refugees, drug and alcohol addicts, female sex workers, and transgender men and women [22].

Cervical cancer screening policies already exist, but combining vaccination and screening –the HPV FASTER strategy– reveals that unvaccinated women are twice as likely to miss screening, meaning that those who are not vaccinated are also not attending screening. Barriers to cervical cancer screening in vulnerable women can be categorised into individual, system, and provider barriers. A study performed in seven European countries [22] identified common individual barriers, such as fear, shame, and low prioritisation of prevention. There are not enough healthcare providers at the provider level. However, different barriers are observed at the system level, making the approach more complex.

Cervical cancer screening programme managers and experts from 27 European countries were asked to identify and rank six high-risk populations considered most vulnerable to cervical cancer and to map existing policies addressing CCS coverage for these groups. Responses from 22 countries consistently identified two at-risk populations: those with low socioeconomic backgrounds and immigrants [22]. However, very few recognised other vulnerable groups, highlighting a gap in awareness and action.

**Fig. 2:**
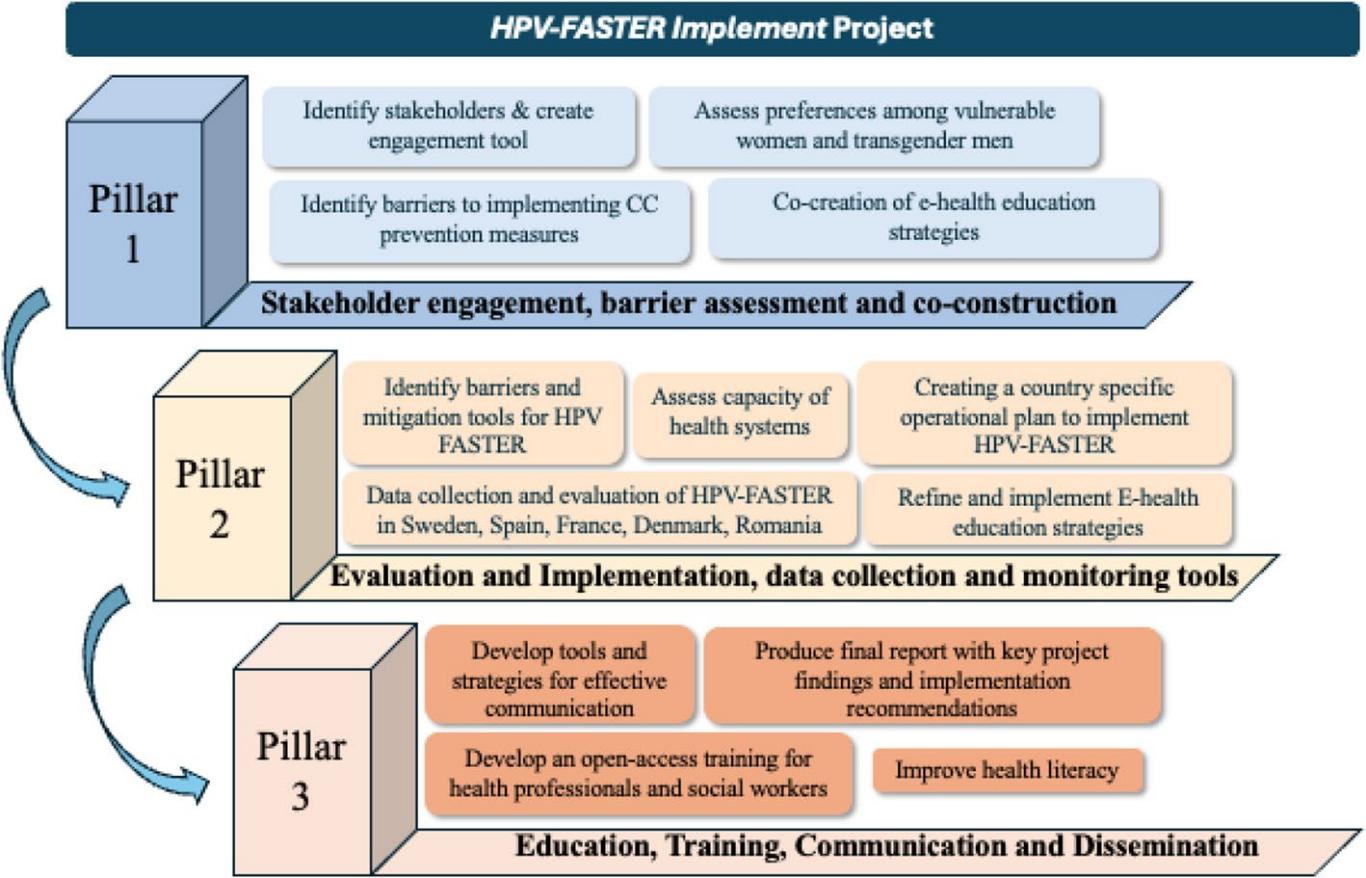
Implementation HPV FASTER in vulnerable populations.Adapted from CBIG Screen project (https://cbig-screen.eu/)

Integrated screening and vaccination of vulnerable groups requires funding, (implementation) research, accessibility, and linkage to care for follow-up after a test-positive result. A strategy to implement and manage HPV FASTER in vulnerable populations has been proposed in Fig. 2. However, its success depends on a genuine political and social commitment to understanding and addressing the needs of these groups to reduce inequity and inequality.

Finally, the Cancer Radar project, coordinated by UMC Amsterdam, aims to quantify the cancer burden among European migrant populations using European cancer registry data. Despite GDPR-related data collection challenges, the aim is to design a data collection tool for collecting fully anonymised data from registries, which will be used to quantify the risk of different forms of cancer in migrant populations stratified by different characteristics. Invitations were sent to 151 registries in 36 countries. So far, 49 responses from 21 countries have been obtained, of which 36 (18 countries) could potentially share data.

Preliminary data from Italy showed a slight increase in cancer incidence and mortality among migrants, potentially due to limited access to healthcare, including cancer screening. Stratification by the number of years spent in the country of origin shows a trend: the longer the period in the country of origin, the higher the risk. While this work focuses on Europe, migration is a global concern. The hope is that methods refined through initiatives like Cancer Radar will ultimately help low- and middle-income countries improve their own screening programmes and better serve vulnerable populations.

## Discussion

Of the three pillars of cervical cancer elimination, vaccination is the strongest. This is evident from the historical success of the EPI programme, initiated in the 1970s. Regardless of economic status, nearly every country has a satisfactory childhood immunisation system. However, the ability to reach adults through screening and treatment is far less developed in most of the world. Currently, a critical constraint for HPV-related cancer control is the infrastructure limitations in the developing world, particularly in delivering effective screening and treatment, which could be mitigated by scaling up HPV vaccination, reducing the future burden and therefore treatment/care demand. While the science is convincing, the implementation remains challenging.

A strategy like HPV EVEN FASTER works well in countries like Sweden, where infrastructure supports high vaccination and screening coverage. In Sweden, the change to school-based HPV vaccination led to 90% coverage, and the switch from cytology to HPV-based screening [in combination with the opportunity for self-sampling/] also led to high coverage, facilitating the launch of the HPV EVEN FASTER concept. However, implementing this in a developing country, where adult screening and treatment infrastructure is lacking, poses significant challenges. It is essential to address the most fundamental needs first before complicating the approach. In countries struggling with infrastructure, focusing solely on HPV vaccination overlooks the women who currently have precancerous lesions or potential invasive cancers. Achieving health equity requires considering the entire population. Building the necessary infrastructure to reach everyone is essential, even if it involves a simplified algorithm, such as a single screening in a lifetime. Building on this framework, mathematical modelling indicates that in regions with moderate to very high cervical cancer burdens, extending vaccination to women up to age 25 should take precedence over vaccinating boys. By contrast, in low-burden environments, catching up older women—those between 26 and 30 years—should be prioritised before offering vaccination to boys. These insights highlight the importance of tailoring preventive interventions to local disease patterns and resources, particularly within low- and middle-income countries where infrastructural constraints can limit the immediate feasibility of large-scale, multicomponent interventions.

The trial and observational data consistently show a significant age effect when vaccinating older women. While adolescents are mostly unexposed to HPV, vaccinating HPV-positive women may offer protection to their partners, though evidence is still lacking. Therefore, the focus should be on vaccinating the young and complementing this with screening for older women.

Infrastructure limitations are a critical issue. The WHO benchmark is to have one radiotherapy unit per million population, but India, for example, only has around 800 radiotherapy units for a population of 1,400 million. Screening is helpful, but taking the programme further will be challenging if the treatment component is missing.

In conclusion, the path toward cervical cancer elimination in LMICs hinges on well-coordinated HPV vaccination strategies tailored to local disease burdens and resource constraints. Prioritising vaccination—particularly through single-dose regimens—can free up resources for wider catch-up campaigns and improved routine coverage, while also bolstering screening and treatment capacity. In practice, approaches must be adapted to national contexts: for instance, India benefits most from catch-up vaccination in females up to age 20, followed by efforts to raise routine vaccination coverage from 50 to 90%. If this coverage target proves elusive, extending protection to boys can help compensate. By contrast, Rwanda achieves the highest dose efficiency by focusing on women aged 26–30. Moreover, combining the nine-valent vaccine with a robust screening programme is more effective than immediately adopting gender-neutral vaccination. Meanwhile, Brazil’s northern regions—where the burden of cervical cancer is disproportionately high—require targeted efforts to improve routine vaccination coverage. Across all settings, addressing inequities is paramount; leveraging existing healthcare touchpoints, such as maternity care, can help ensure broader reach and engagement. Ultimately, achieving the WHO’s recommended 90% vaccination coverage, and 70% screening coverage will be essential for eliminating cervical cancer in LMICs and realising equitable health outcomes for women worldwide.

### Lessons learned

The global cervical cancer incidence is 14 per 100,000 women, with a mortality rate of 7 per 100,000 women. The WHO elimination target is to reduce the incidence to 4 per 100,000 women. However, for LMICs it is very challenging to reach the 70% screening target. Vaccinating women up to the age of 40 or 45 may partially solve this problem, provided that empirical evidence demonstrates a reduction in cervical cancer risk, as it will require less screening and treatment afterwards. However, the HPV (EVEN) FASTER approach may lead to equity issues, with the use of the vaccine in low-risk adult women in HIC instead of girls in high-burden LMICs. The efficacy and effectiveness of the vaccine are high in young children but much lower in older women. Therefore, the priority should still be to vaccinate young girls.

The WHO target, based on age-standardised incidence rates, places significantly higher value on preventing cancers in women under 30 years, despite their rarity. A standardised lifetime risk under maximum life expectancy may be a more attractive elimination target but requires context-specific modelling. Data from Sweden suggest that an aspirational elimination target could be to reduce the standardised lifetime risk to around 1 in 917 women. This risk is not fixed over time as life expectancy goes up.

Regardless of how one looks at the most efficient way to apply vaccination to prevent cervical cancer, results depend on the burden, the time since the start of the vaccination programme, and the vaccination coverage achieved. In all cases, multi-age cohorts and increased vaccination coverage in the routine cohort are more efficient than gender-neutral vaccination. Results are similar for LMICs with a moderate to very high burden. In countries with a low burden, it is more efficient to vaccinate older women (over the age of 30) than boys. Waning of the immune response has no impact on the order of efficiency, suggesting that women are protected during the period they are most likely to acquire HPV infections.

Screening policies appear to have plateaued after showing clear initial benefits. Risk-based screening, focusing on vulnerable groups, may help eliminate disparities. This approach involves looking at individual and contextual barriers and developing political and societal knowledge and understanding of vulnerable populations and the difficulties they face in their prevention efforts. An HPV-FASTER-like programme can help to provide equity to vulnerable populations. By measuring the effectiveness in preventing cervical cancers in vulnerable populations and the additional economic resources needed, the benefits-to-harm ratio of the HPV FASTER over the standard of care can be calculated. Furthermore, innovative screening approached based on point-of-care testing are being investigated for underserved populations (migrants, refugees, internally displaced population and hard to reach population).

### Way forward

The best solution for acceleration is context-specific, as one size does not fit all. Political will and implementation research are needed to adapt the general concepts and results from modelling to specific country contexts. To this end, WHO has developed an implementation agenda for global use. Real-time data are expected to lead to more flexible problem-solving.

#### Vaccination

Achieving equitable cervical cancer control between HICs and LMICs depends on meeting ambitious targets, notably 90% vaccination coverage—using multi-age cohort (MAC) and gender-neutral approaches—and 70% screening coverage. While vaccines offer substantial value, and the at-risk population is generally well-defined, strategies for effectively reaching these groups are not always straightforward. Out-of-school adolescents, for instance, pose a particular challenge, suggesting that lowering the age of vaccination may improve coverage. In addition, cost remains a significant barrier; however, the introduction of new vaccines developed in China and India could help reduce expenses, enabling broader vaccination and thereby curbing transmission.

#### Screening

In many LMICs, conducting cervical cancer screening presents a far greater challenge than administering vaccines, largely because each setting has unique needs. Yet screening must be paired with prompt access to treatment—making it unethical as there is little benefit in detecting abnormalities if patients who test positive cannot receive adequate care. Unfortunately, both surgical and radiotherapy options remain limited in these regions due to shortages of skilled personnel and resources, underlining the urgent need for more trained healthcare workers.

Maternity care frequently serves as one of the few consistent points of contact that many women have with the healthcare system, as well as HIV care for people living with HIV, providing an opportune moment to offer screening or even vaccination. Looking ahead, the introduction of new point-of-care tests may simplify the screening process in resource-limited areas, thereby increasing the likelihood of early detection and treatment.

## Conclusion

While progress is being made to reach the WHO cervical cancer elimination targets, further acceleration is essential. Strategies such as HPV FASTER and HPV EVEN FASTER are technically feasible and offer potential value. However, given the global distribution of the burden of cervical cancer and the limitations of resources, alternative approaches such as transitioning to single-dose vaccination, boosting vaccination coverage, and implementing multi-age cohort vaccination up to age 20 are likely to be more impactful and equitable. These strategies should be prioritised to meet the WHO cervical cancer elimination timeline. The expected availability of additional vaccines and POC tests should help speed up both vaccination and screening globally.

## Data Availability

All the presentations of the meeting report are published on the website (www.hpvboard.org) after speakers’ approval.

## Abbreviations

2vHPV: Bivalent HPV Vaccine
4vHPV: Quadrivalent HPV Vaccine
9vHPV: Nonavalent HPV Vaccine
AI: Artificial Intelligence
ASR: Age-Standardised Rate
CIN: Cervical Intraepithelial Neoplasia
DALY: Disability-Adjusted Life Year
EPI: Expanded Programme on Immunisation
FVU: First Void Urine
GDPR: General Data Protection Regulation
HICs: High-Income Countries
HLA: Human Leukocyte Antigen
HPV: Human Papillomavirus
JoRRP: Juvenile-Onset Recurrent Respiratory Papillomatosis
LMICs: Low- and Middle-Income Countries
MAC: Multiple-Age Cohort
NNV: Number Needed to Vaccinate
POC: Point-of-Care
ProMetHeoS: Progression Model of The HPV Infection
R0 (R_0_): Basic Reproduction Number
RISCC: Risk-Based Screening for Cervical Cancer
RRP: Recurrent Respiratory Papillomatosis
SWOT: Strengths, Weaknesses, Opportunities, and Threats
TPPs: Target Product Profiles
WHO: World Health Organization
